# The association of elevated maternal genetic risk scores for hypertension, type 2 diabetes and obesity and having a child with a congenital heart defect

**DOI:** 10.1371/journal.pone.0216477

**Published:** 2019-05-29

**Authors:** Michelle Kaplinski, Deanne Taylor, Laura E. Mitchell, Dorothy A. Hammond, Elizabeth Goldmuntz, A. J. Agopian, the Pediatric Cardiac Genomics Consortium

**Affiliations:** 1 Department of Pediatrics, Division of Cardiology, Children’s Hospital of Philadelphia, Philadelphia, Pennsylvania, United States of America; 2 Department of Biomedical and Health Informatics, Children’s Hospital of Philadelphia, Philadelphia, Pennsylvania, United States of America; 3 Department of Pediatrics, Perelman School of Medicine, University of Pennsylvania, Philadelphia, Pennsylvania, United States of America; 4 Department of Epidemiology, Human Genetics and Environmental Sciences, UTHealth School of Public Health, Houston, Texas, United States of America; Centro Cardiologico Monzino, ITALY

## Abstract

**Background:**

Maternal hypertension, type 2 diabetes (T2D) and obesity are associated with an increased risk of having offspring with conotruncal heart defects (CTDs). Prior studies have identified sets of single nucleotide polymorphisms (SNPs) that are associated with risk for each of these three adult phenotypes. We hypothesized that these same SNPs are associated with maternal risk of CTDs in offspring.

**Methods and results:**

We evaluated the parents of children with a CTD ascertained from the Children’s Hospital of Philadelphia (n = 466) and by the Pediatric Cardiac Genomic Consortium (n = 255). We used a family-based design to assess the association between CTDs and the maternal genotype for individual hypertension, T2D, and obesity-related SNPs and found no association between CTDs and the maternal genotype for any individual SNP. In addition, we calculated genetic risk scores (GRS) for hypertension, T2D, and obesity using previously published GRS formulas. When comparing the GRS of mothers to fathers, there were no statistically significant differences in the mean for the combined GRS or the GRS for each individual condition. However, when we categorized the mothers and fathers of cases with CTDs as having high (>95^th^ percentile) or low (≤95^th^ percentile) scores, compared to fathers, mothers had almost two times the odds of having a high GRS for hypertension (OR 1.7, 95% CI 1.0, 2.8) and T2D (OR 1.8, 95% CI 1.1, 3.1).

**Conclusions:**

Our results support a link between maternal genetic risk for hypertension/T2D and CTDs in their offspring. These associations might be independent of maternal phenotype at conception.

## Introduction

Congenital heart defects are the most common, significant congenital abnormality in newborns. They occur in approximately 1% of all live births and 10% of stillbirths. Little is known regarding the etiology of these defects, but both genetic and environmental factors have been identified [1]. Conotruncal defects (CTDs) are among the most common types of congenital heart defects, accounting for one fifth of all heart defects [2,3]. Although 22q11.2 deletions, trisomy 21 and other genetic syndromes are seen in nearly 25% of cases with CTD defects, the etiology in most cases remains unknown. Given the high recurrence rate and heritability in families (approximately 4–5% recurrence risk for any CTD in siblings) [4], these unexplained CTDs are nonetheless thought to have a genetic basis [5, 6].

Major risk factors for congenital heart defects, and CTDs specifically, include maternal hypertension (HTN), type 2 diabetes (T2D), and obesity [7–10]. In fact, mothers who have HTN prior to pregnancy have been found to have an increased risk of having a child with a CTD, even after controlling for medication use [7,9]. Diabetic or obese mothers also have increased risk for these defects in offspring [8,10,11]. Based on the relatively high frequency of these conditions in females of reproductive age in the general population (i.e., ~25% for obesity [12], 5% for T2D [13] and 5% for chronic HTN [14] and the reported magnitudes of association between these maternal conditions and the risk of CTDs (i.e., OR~1.3 [15], 5.4 [16] and 1.3 [9,17] respectively), these maternal conditions may account for a substantial proportion of the risk for CTDs in offspring.

The specific mechanisms underlying the associations between these common chronic maternal conditions and congenital heart defects are unclear, and could involve abnormal blood flow to the placenta (e.g. HTN) [9,18,19], glucose metabolism (T2D, obesity) [16,20], or other pathways. Even in the absence of the overt maternal phenotype, a genetic predisposition to one of these conditions in the mother might be associated with CTD risk to her offspring due to a more subtle alteration of the embryonic environment (e.g., sub-clinical maternal phenotype). An association between maternal genetic variation and offspring risk (independent of the offspring’s genotype) is known as a maternal genetic effect. These effects can be viewed as resulting from the influence of the maternal *in utero* environment, caused by maternal genetic variation as opposed to inheritance of these genes by the infant [21]. A better understanding of associations between maternal genotypes and the risk to offspring could help identify phenotypically normal, yet genotypically high-risk mothers. This could aid in increased screening for high-risk pregnancies (and subsequent early diagnoses and options for early interventions) and improved pre-pregnancy counseling.

Several large-scale genome wide association studies have identified single nucleotide polymorphisms (SNPs) associated with HTN, T2D and obesity [22–26]. As compared to individual SNPs, select groups of SNPs have collectively been found to be associated with these conditions in adults; together these groups of SNPs have been used to predict risk for these three conditions by way of a genetic risk score [22,27]. We therefore hypothesized that pregnant women with elevated genetic risk scores for these chronic conditions were more likely to have a child with a CTD as compared to women with lower scores. As proof of this concept, a recent study demonstrated that women with high maternal genetic risk scores for HTN, T2D and obesity were more likely to have an infant that was either large or small for gestational age as compared to mothers with lower genetic risk scores, independent of maternal phenotype [27]. To test our hypothesis, we evaluated the maternal genetic risk scores and the individual component SNPs for HTN, T2D and obesity to determine whether the maternal genetic risk for these conditions was associated with the risk of having a child with a CTD.

## Methods and materials

### Study population

The study population included two independent cohorts of CTD case-parent trios recruited from the Children’s Hospital of Philadelphia (CHOP) and the Pediatric Cardiac Genomic Consortium (PCGC) [28]. There were 466 case-parent trios recruited at CHOP and 255 case-parent trios by the PCGC. Details of ascertainment and recruitment have been described previously [29,30]. Patients with genetic syndromes were excluded.

Briefly, the CHOP CTD trios were recruited from The Cardiac Center at CHOP from 1992 to 2010. Individuals of all races/ethnicities were eligible to participate, but for our analyses, we restricted to Caucasian trios. All subjects provided informed consent under a protocol approved by the Children’s Hospital of Philadelphia Institutional Review Board for the Protection of Human Subjects.

The PCGC trios were recruited from 2010 to 2012. Five main clinical centers participated: Harvard Medical School (Boston Children’s Hospital and Brigham and Women’s Hospital), Yale School of Medicine, Columbia University Medical Center, Icahn School of Medicine at Mount Sinai, and the Children’s Hospital of Philadelphia as well as four satellite clinical sites at the University of Rochester Medical Center, Cohen Children’s Medical Center, Children’s Hospital of Los Angeles, and University College London. For both the CHOP and PCGC cohorts, the cases had one of the following CTD cardiac defects: tetralogy of Fallot, D-transposition of the great arteries, ventricular septal defects (conoventricular, posterior malalignment and conoseptal hypoplasia), double outlet right ventricle, isolated aortic arch anomalies, truncus arteriosus or interrupted aortic arch. There was no overlap between those patients ascertained at CHOP and those recruited at CHOP for the PCGC. We limited our analyses to non-Hispanic Caucasian families.

### Data collection

For both cohorts, case demographics, cardiac diagnoses, and information on extracardiac defects were collected through structured electronic or paper case report forms [29,30]. Specifically, data were obtained on case CTD type, race and extracardiac defects; father’s race and age; and maternal race, age, pre-pregnancy body mass index (BMI), pre-gestational diabetes and gestational diabetes.

All cardiac diagnoses were obtained from echocardiographic or other imaging modalities and diagnoses were confirmed by a pediatric cardiologist. Extracardiac defects were confirmed by review of medical records, including available genetic consultation records. Parental information was obtained through family interviews or medical record review. Additional details about the pregnancy (i.e. preeclampsia and HTN medication use) were collected from the case’s parents in all CHOP cases and in cases from the PCGC if the case was enrolled at age <1 year [30]. Information was available on maternal HTN medication use during pregnancy from both CHOP and PCGC subjects, but mothers were not directly asked about HTN during pregnancy in the CHOP cohort.

### Descriptive statistics

We tabulated counts and percentages for maternal, paternal and offspring demographic information separately for the CHOP and PCGC cohorts. For continuous variables, we tested for normality using the Shapiro-Wilk test and we log-transformed the data when appropriate. Differences in the distribution of categorical variables in the CHOP and PCGC cohorts were tested using Fisher’s exact test and differences in the mean values of continuous variables were assessed using a Student’s t test. These analyses were performed using Stata 13 (Stata Corp, College Station, TX). A P-value ≤0.05 was considered significant, unless otherwise specified.

### Genotyping

All study subjects provided either blood or saliva samples, from which DNA was extracted using standard methods, as previously described [28]. Genotyping, imputaton and quality control procedures have been described. Briefly, due to differences in the timing of the availability of data from CHOP and PCGC, subsets of data from each of these two sources were genotyped in separate batches on different arrays (CHOP: 550K, 610K; PCGC: 1M and 2.5M). In addition, data from CHOP and PCGC were imputed separately, using genotype data only for the SNPs that overlapped the relevant genotyping platforms (e.g. for CHOP, the overlap of 550K and 610K). Both sets of imputations (i.e. CHOP and PCGC) were performed using IMPUTE2 and reference data from the 1000 Genomes Project.

### Individual SNP analyses

We previously performed SNP-level, trio-based maternal effect genome wide association studies (GWAS) to assess the association of the maternal genotype for individual SNPs with CTDs. We conducted these analyses separately in the CHOP and PCGC cohorts [28], using a multinomial likelihood approach [31] implemented in the EMIM software package [29,32]. A meta-analysis of the results from the CHOP and PCGC cohorts was conducted using GWAMA [29].

For the current study, we used p-values from the meta-analysis to assess the association between common (minor allele frequency >0.05) maternal genotypes for individual SNPs in HTN, T2D and obesity-related genes. Specifically, we assessed maternal genotypes for SNPs included in published genetic risk scores for HTN (31 SNPs), obesity (30 SNPs) and T2D (46 SNPs). Significance was assessed using a Bonferroni correction for the number of SNPs evaluated for each condition (i.e. p<0.002 for HTN and obesity; p<0.001 for TD2). SNPs with association p-values <0.05, but greater than the Bonferroni adjusted p-values were considered “suggestive.”

### Genetic risk score analyses

Following the single SNP analysis, we calculated genetic risk scores in mothers and fathers using the raw genotype data from both cohorts (PCGC and CHOP) based on the approach of Tyrrell et al [22,27]. A separate risk score was computed for each condition (HTN, obesity and T2D). Data for 31 of 33 SNPs comprising the HTN genetic risk score, 46 of 55 T2D SNPs and all 30 of the obesity SNPs were available for this analysis (S1 Table) [27]. There was no overlap between any of the SNPs among the three different genetic risk scores. Briefly, each score was determined as the sum of the number of at-risk alleles for each SNP (0, 1, or 2), weighted by the magnitude of the association with the given maternal condition. Both the definition of the at-risk allele and the weighting factor were as defined by Tyrrell et al.

We derived genetic risk scores for each of the three conditions separately and also created a combined genetic risk score that represented the sum of the risk scores for each of the three conditions. Because, to our knowledge, methods designed for directly assessing associations with maternal genetic risk scores do not exist for trio-based data, we used a case-control design for our risk score analyses, whereby mothers served as “cases” and fathers served as “controls.” Thus, in the absence of maternal genetic effects, mothers and fathers would have similar genetic risk scores, whereas higher risk scores among mothers than fathers would suggest maternal genetic effects. We evaluated differences in the mean genetic risk scores of mother and fathers as continuous variables using the Student’s t test. We also considered genetic risk scores as categorical variables, to determine whether there was a threshold effect, such that a maternal genetic effect would only be evident in mothers with very elevated genetic risk scores. Since no data exists on what that threshold value would be, we considered three categorization schemes, based arbitrarily on cut-points at the top 5^th^, 10^th^ and 25^th^ respective percentiles of the genetic risk score distribution in fathers. Thus, we used a Fisher’s exact test to assess differences in the distribution of mothers and fathers for each of these categories of elevated genetic risk scores (top 5^th^, 10^th^ and 25^th^). Although our main analyses were conducted among the full analytic group (CHOP and PCGC pooled data), we also repeated all of the above tests in *post-hoc* subgroup analyses of the CHOP and PCGC cohorts separately, to see if either individual cohort was driving the observed associations.

We also repeated the genetic risk score comparisons in *post-hoc* sub–analyses among the full analytic group after removing trios with mothers who had preeclampsia, hypertension (treated with medication), or pregestational diabetes. In addition, we separately evaluated those trios with infants with tetralogy of Fallot, as that was our largest conotruncal type in both the PCGC and CHOP cohorts. In both of these analyses, we compared the top 5^th^ percentile of genetic risk scores in mothers versus fathers and also compared the mean genetic risk score.

## Results

### Baseline characteristics

We evaluated the parents of children with a CTD ascertained from the Children’s Hospital of Philadelphia (n = 466) and by the Pediatric Cardiac Genomic Consortium (n = 255). The characteristics of mothers, fathers and cases for each cohort are presented in Table 1. Significantly more women reported having preeclampsia in the PCGC cohort as compared to the CHOP cohort (7.0% versus 1.0%, respectively, p-value <0.01). In addition, there were significantly fewer cases with extracardiac defects in the PCGC cohort than in the CHOP cohort (31.1% versus 49.8%, respectively, p-value <0.01). There was also a significant difference in the distribution of the subtypes of CTDs between the two cohorts (p-value <0.01). For instance, compared to the PCGC cohort, the CHOP cohort had a higher proportion of cases with tetralogy of Fallot (41% versus 32%) and a lower proportion with L-transposition of the great arteries (0% versus 4.9%).

**Table 1:** Characteristics of conotruncal heart defect (CTD) cases and their parents in the CHOP and PCGC by study cohorts.

	PCGC N (%) N = 225*	CHOP N (%) N = 466	P values
Case CTD categories †			
Isolated aortic arch anomaly	8 (3.6)	22 (4.7)	<0.01
D-Transposition of the great arteries	42 (18.9)	93 (20.0)	
Double outlet right ventricle	32 (14.3)	48 (10.3)	
Interrupted aortic arch	6 (2.7)	6 (1.3)	
Tetralogy of Fallot	72 (32.3)	190 (40.8)	
Truncus arteriosus	7 (3.1)	13 (2.8)	
Ventricular septal defect	36 (16.1)	88 (18.8)	
Right ventricle-aorta/Pulmonary atresia	1 (0.4)	1 (0.2)	
L-Transposition of the great arteries	11 (4.9)	0 (0.0)	
Complex CTD	8 (3.6)	5 (1.1)	
Case sex			
Male	138 (61.0)	284 (61.0)	0.93
Female	87 (39.0)	182 (39.0)	
Case extracardiac malformations‡			
Yes	70 (31.1)	232 (49.8)	<0.01
No	155 (68.9)	226 (48.5)	
Unknown	0 (0.0)	8 (1.7)	
Maternal race			
White	223 (99)	466 (100)	0.13
Asian	1 (0.5)	0 (0)	
More than one race	1(0.5)	0 (0)	
Paternal race			
White	223 (99)	466 (100)	0.13
Hispanic	1 (0.5)	0 (0)	
Unknown	1 (0.5)	0 (0)	
Maternal age			
<20	4 (2.0)	7 (2.0)	0.92
20-<25	24 (11.0)	45 (10.0)	
25-<30	52 (23.0)	114 (26.0)	
30-<35	91 (41.0)	166 (38.0)	
35-<40	46 (20.0)	88 (20.0)	
>40	6 (3.0)	16 (4.0)	
Paternal age			
<20	3 (1.0)	1 (1.0)	0.30
20-<25	13 (6.0)	27 (6.0)	
25-<30	49 (22.0)	96 (23.0)	
30-<35	74 (33.0)	165 (39.0)	
35-<40	61 (28.0)	96 (23.0)	
>40	22 (10.0)	35 (8.0)	
Maternal BMI			
<18.5	12 (6.0)	11 (4.0)	0.10
18.5-<25	119 (57.0)	187 (68.0)	
25-<30	50 (24.0)	47 (17.0)	
>30	28 (13.0)	31 (11.0)	
Maternal pregestational diabetes			
Yes	2 (1.0)	3 (1.0)	0.11
No	221 (98.0)	463 (99.0)	
Unknown	2 (1.0)	0 (0.0)	
Maternal gestational diabetes			
Yes	13 (6.0)	21 (4.0)	0.39
No	209 (93.0)	432 (93.0)	
Unknown	3 (1.0)	13 (3.0)	
Maternal preeclampsia			
Yes	6 (7.0)	4 (1.0)	<0.01
No	76 (93.0)	449 (96.0)	
Unknown	0 (0.0)	13 (3.0)	
Maternal HTN med during pregnancy			
Yes	2 (2.0)	5 (1.0)	0.10
No	80 (98.0)	440 (94.0)	
Unknown	0 (0.0)	21 (5.0)	

CHOP indicates Children’s Hospital of Philadelphia; PCGC, Pediatric Cardiac Genomics Consortium; CHD, congenital heart defect; CTD, conotruncal defect; BMI, body mass index; HTN med, hypertension medication

* The study was designed to be limited to Caucasian trios

† The numbers for each variable may not sum to the total due to missing information

‡ Any major abnormality outside of cardiac defect, including dysmorphic features

### Single SNP results

We used a family-based design to assess the association between CTDs and the maternal genotype for individual hypertension, T2D, and obesity-related SNPs from previously performed SNP-level, trio-based maternal effect genome wide association studies (GWAS) [28,29]. After accounting for multiple comparisons, no single SNP in our analyses was significantly associated with CTDs (S2 Table). A few SNPS had p-values suggestive of associations, including one obesity-related SNP, [rs2815752 (*NEGR1*), unadjusted p = 0.004] and several HTN-related SNPs [rs13139571 (*GUCY1A3-GUCY1B3*), rs11191548 (*CYP17A1-NT5C2*) and rs1801253 (*ADRB1*), each with unadjusted p = 0.01] (Table 2).

**Table 2:** Results of single SNP analysis: SNPs with suggested association (p<0.05).

Nearest Gene	SNP	Location	P value
Hypertension-related SNPs*			
GUCY1A3	rs13139571	Intron	0.01
CYP17A1-NT5C2	rs11191548	Non coding variant	0.01
ARHGAP42	rs633185	Intron	0.03
ADRB1	rs1801253	Exon	0.01
Obesity-related SNPs †			
NEGR1	rs2815752	Intron	0.004
KCTD15	rs29941	Non coding variant	0.04
Diabetes-related SNPs ‡			
HNF4A	rs4812829	Intron	0.03
MAEA	rs6819243	Intron	0.03

*SNP indicates single nucleotide polymorphism; GWAS, genome wide association study

†Bonferroni significance p-value threshold ≤ 0.002 (30 obesity SNPs, 31 HTN SNPs)

‡Bonferroni significance p-value threshold ≤0.001 (46 T2D SNPs)

### Genetic risk score results

We calculated genetic risk scores (GRS) for hypertension, T2D, and obesity for the mothers and fathers using previously published GRS formulas [27]. Our main analyses were performed among the full analytic group (i.e., pooled CHOP and PCGC cohort data). There were no statistically significant differences in the mean for the combined genetic risk scores (HTN+T2D+obesity scores) between mothers (mean score: 26.0) and fathers (mean score: 25.8) (Table 3). Similarly, there was no significant difference between the mothers’ and fathers’ mean genetic risk score for each individual condition (i.e., HTN, T2D and obesity) (Table 3).

**Table 3:** Comparison of mothers' versus fathers' genetic risk scores (GRS) for adult conditions.

GRS Type	Mean *	Mean P-value †	95^th^ %ile OR (95% CI)‡	Mean	Mean P-value	95^th^ %ile OR (95% CI)	Mean	Mean P-value	95^th^ %ile OR (95% CI)
	Full cohort analysis			PCGC subgroup analysis				CHOP subgroup analysis		
Combined § (107 SNPs)	Mothers’: 26.0 Fathers’: 25.8	0.24	1.78 (1.10, 2.94)	Mothers’: 26.2 Fathers’: 25.8	0.03	2.74 (1.23, 6.54)	Mothers’: 25.8 Fathers’: 25.8	0.96	1.35 (0.71, 2.60)
Obesity (30 SNPs)	Mothers’: 3.8 Fathers’: 3.8	0.79	1.54 (0.94, 2.56)	Mothers’: 3.9 Fathers’: 3.8	0.12	2.84 (1.17, 7.57)	Mothers’: 3.8 Fathers’: 3.8	0.41	1.1 (0.60, 2.00)
HTN (31 SNPs)	Mothers’: 17.8 Fathers’; 17.7	0.29	1.70 (1.04, 2.83)	Mothers’: 17.9 Fathers’: 17.6	0.08	2.49 (1.10, 6.01)	Mothers’: 17.7 Fathers’: 17.7	0.94	1.35 (0.71, 2.60)
Type II Diabetes (46 SNPs)	Mothers’: 4.4 Fathers’: 4.4	0.64	1.84 (1.12, 3.07)	Mothers’: 4.4 Fathers’: 4.4	0.81	1.86 (0.85, 4.25)	Mothers’: 4.4 Fathers’: 4.4	0.63	1.86 (1.00, 3.69)

GRS indicates genetic risk score; SNPs, single nucleotide polymorphisms; OR, odds ratio; CI, confidence interval

*Mean GRS of mothers’ and fathers’ for each adult condition

†P-value for comparison between mothers’ mean GRS to fathers’ mean GRS for each adult condition

‡ Categorical analysis of the number of mothers versus the number of fathers that have a GRS above the 95^th^ percentile, based on the fathers’ score. The 95^th^ percentile cutoffs for the GRS for each condition are as follows: Combined SNPs GRS cutoff, 28.9; Obesity SNPs GRS cutoff, 4.6; Hypertension SNPs GRS cutoff, 20.4; Type II Diabetes SNPs GRS cutoff, 5.0

§ Combined genetic risk scores for body mass index, hypertension and type 2 diabetes

When the genetic risk scores were analyzed as dichotomous variables, where mothers and fathers of cases with CTDs were categorized as having either high (>95^th^ percentile) or low (≤95^th^ percentile) scores, mothers had almost two times the odds of having a high versus low combined genetic risk score as compared to fathers (OR 1.78, 95% confidence interval [CI] 1.1, 2.94). Mothers also had almost two times the odds of having a high versus low genetic risk score compared to fathers for both HTN (OR 1.7, 95% CI 1.0, 2.8) and T2D (OR 1.8, 95% CI 1.1, 3.1). Although mothers also had increased odds of having a high versus low obesity genetic risk score, the difference was not statistically significant (OR 1.5, 95% CI 0.9–2.6). Similar results were found when segregating the top 10^th^ percentile from the lower 90^th^ percentile using the methods described; however, no associations were detected when comparing the top 25^th^ percentile to the lower 75^th^ percentile (S3 and S4 Tables).

To further understand these associations in the combined cohort, we performed sub-analyses within each separate cohort (i.e., PCGC and CHOP). Among the PCGC subgroup, results were similar to those among the full analytic group, although the associations with all four genetic risk scores were stronger, and the association with the obesity genetic risk score became significant in the categorical analysis (OR 2.8, 95% CI 1.2, 7.6) (Table 3). Among the CHOP subgroup, results for T2D were similar to those among the full analytic group, but the associations with the HTN and the combined genetic risk scores were no longer statistically significant (Table 3). However, the direction of association with each of the four genetic risk scores were similar (i.e. OR>1) across the CHOP and PCGC cohorts.

To address the extent to which the corresponding overt maternal phenotypes may have contributed to risk, we conducted a sub-analysis, excluding mothers from the full cohort who had preeclampsia, hypertension (treated with medication), or pregestational diabetes. The results from this sub-analysis were similar to our main results (data not shown). BMI was not considered in this sub-analysis, due to accuracy concerns related to the observed BMI distribution. These results suggest that effects of hypertension and diabetes genetic risk scores were independent of the corresponding overt maternal phenotypes.

When we analyzed trios with infants with tetralogy of Fallot, the magnitude of association with the hypertension genetic risk score was larger (OR: 3.6, 95% CI: 1.4–9.5) and the other magnitudes of association were similar or attenuated compared to those seen in the full cohort (Table 4).

**Table 4:** Sub-analysis in trios with a child with tetralogy of Fallot. Comparison of mothers' versus fathers' genetic risk scores (GRS) for adult conditions.

GRS Type	Mean *	Mean p-value †	95^th^ %ile OR (95% CI)‡
Combined § (107 SNPs)	Mothers’: 26.0 Fathers’: 25.7	0.06	2.58 (1.04, 6.43)
Obesity (30 SNPs)	Mothers’: 3.8 Fathers’: 3.8	0.88	1.73 (0.82, 3.64)
HTN (31 SNPs)	Mothers’: 17.9 Fathers’; 17.5	0.04	3.64 (1.39, 9.54)
Type II Diabetes (46 SNPs)	Mothers’: 4.4 Fathers’: 4.4	0.46	1.22 (0.64, 2.31)

GRS indicates genetic risk score; SNPs, single nucleotide polymorphisms; OR, odds ratio; CI, confidence interval

*Mean GRS of mothers’ and fathers’ for each adult condition.

†P-value for comparison between mothers’ mean GRS to fathers’ mean GRS for each adult condition

‡ Categorical analysis of the number of mothers versus the number of fathers that have a GRS above the 95^th^ percentile, based on the fathers’ score. The 95^th^ percentile cutoffs for the GRS for each condition are as follows: Combined SNPs GRS cutoff, 28.9; Obesity SNPs GRS cutoff, 4.6; Hypertension SNPs GRS cutoff, 20.4; Type II Diabetes SNPs GRS cutoff, 5.0.

§ Combined genetic risk scores for body mass index, hypertension and type 2 diabetes

## Discussion

In this study, we tested whether a maternal genetic predisposition for adult conditions that are themselves associated with CTD in offspring is also associated with CTDs in offspring. We used published genetic risk scores for these adult conditions to focus our analysis on known, risk-related variants. Our results suggest that maternal genetic risk for common chronic adult conditions may be associated with the risk of having a child with a CTD. In particular, mothers had an increased odds of having a higher genetic risk score for HTN and T2D as compared to fathers when the parents were dichotomized into high and low subgroups. There was no difference between the mean maternal and paternal genetic risk score for each condition. Thus, our findings suggest that mothers with the highest genetic risk scores (i.e., >90th percentile) may be at increased risk for having affected offspring due to a maternal genetic effect, though the exact threshold of the genetic risk score remains to be defined.

We combined data from the CHOP and PCGC cohorts to augment power to detect a genetic association, based on the assumption that the two were very similar cohorts, with similar case phenotype definitions, as well as similar case ascertainment, family recruitment, and genotyping methods. However, the two groups were ascertained sequentially (CHOP:1992–2010; PCGC: 2010–2012) and in somewhat diverse geographic locations and there were differences in the prevalence of extracardiac defects and distribution of cardiac phenotypes in the cases. These differences may, at least in part, explain any observed variation in the magnitudes of the odds ratios across the two groups. For example, it may be that a maternal genetic predisposition for common adult conditions may increase risk for only certain types of CTD defects. Our tetralogy of Fallot sub-analysis may partially support this notion, as the magnitude of association with the hypertension genetic risk score was higher compared to the corresponding association with all CTDs. Future work focusing on specific sub-phenotypes may be worthwhile.

Not surprisingly, a combination of disease-risk SNPs represented by the genetic risk score rather than individual SNPs were associated with disease risk for affected offspring. This observation is consistent with the known genetic complexity of disease risk for CTDs. Although these genetic risk scores are the most predictive for the adult conditions, we do not know whether a different subset of SNPs would be associated with an even greater risk for CTDs in offspring. Further, the maternal phenotype for each of these conditions, be it overt or subclinical, in conjunction with genetic predisposition, might modify disease risk to offspring. When we repeated our analysis removing mothers with overt preeclampsia, hypertension (treated with medication) and pregestational diabetes, we saw no difference in our results, which suggests that the overt maternal phenotype was likely not solely responsible for our findings.

All three maternal chronic diseases studied here (HTN, T2D, and obesity) have been associated with congenital heart defects in epidemiologic studies, but the mechanisms underlying these association are undetermined [13,15,16,33,34]. Many studies have theorized that maternal HTN causes changes in blood flow to the uterus during pregnancy, which results in abnormal cardiac development, based on the fact that medications that lead to hypotension, such as ACE inhibitors, have been associated with cardiac defects [9,35,36]. Yet, no animal studies have been performed to demonstrate an association between changes in blood flow to the uterus due to maternal hypertension and cardiac defects in the fetus [9,18,19]. Alternatively, several animal studies have shown that epigenetic modifications caused by an adverse prenatal environment (e.g. maternal HTN) may affect angiogenesis and placental growth, which are processes that influence fetal cardiac development [33,34,37]. It could be hypothesized that it is not only the phenotypic effect of hypertension causing abnormal flow, but rather an independent genetic trigger, that increases risk for abnormal fetal cardiac development.

Similarly, mothers with a genetic predisposition for chronic adult diseases may be susceptible to subclinical changes in glucose, adipose production and vascular development in the placenta. Such changes might alter fetal cardiac development, which would help to explain the associations with maternal obesity and diabetes. In fact, mild derangements in lipid production and hyperglycemia in early embryogenesis have been shown to alter expression of the genes of the offspring in the developing fetal heart, increasing risk for congenital heart disease [16,20]. These observations may suggest complex interactions are at play between the maternal genotype, maternal phenotype, and the offspring genotype. While our estimates of individual SNPs accounted for both maternal and offspring genes, our data and study design did not allow for a formal evaluation of all of these complex main effects and interactions (discussed further below), and a better delineation of the underlying mechanisms involved in genetic risk is needed.

Maternal genotypes have been associated with CTDs and other congenital heart defects in the fetus. For example, previous studies have demonstrated a possible association between maternal variants in *SLC22A24* and *MTHFR* and having a child with a CTD[28,38–40]. It is likely that additional maternal genetic risk factors remain to be identified. Further, the concept of maternal genetic predispositions to common chronic adult diseases affecting the offspring has been demonstrated previously. A recent study found that these same maternal genes and genetic risk scores we evaluated were also associated with infant birth weight [27]. Thus, our results may help elucidate maternal genetic pathways that increase risk for a variety of adverse phenotypes in offspring.

Limitations to the study include limited phenotypic data on the mothers ascertained during their pregnancy. However, very few mothers reported using HTN medications during pregnancy (N = 7) or having pregestational diabetes (N = 5). We also repeated our analysis after removing these trios, and had similar findings. Unfortunately, we were unable to control for offspring genotype when evaluating the genetic risk scores because there are no statistical tools currently available to analyze multiple weighted SNPs using a trio-based design [32]. However, if the results were only due to the offspring genotype, the parental genotypes would contribute only as a result of the alleles transmitted to the child, and the parental mating combinations would be expected to be symmetrical. In addition, data on smoking and alcohol use were not consistently collected for both groups (e.g., many subjects were recruited well after the pregnancy). Thus, we did not have the necessary data to evaluate for potential effect modification of the maternal genetic risk scores by smoking or alcohol use. Major strengths of our study include use of a relatively large study sample for evaluating congenital heart defects and evaluation of both single and group-level effects of SNPs.

## Conclusions

We found that groups of T2D and HTN-related SNPs in the mother may be associated with risk for CTDs in offspring. This aids in our understanding of the complex genetic mechanisms that underlie the established associations between the presence of these overt maternal conditions and heart defects in children.

## Supporting information

S1 Checklist Strega checklist. (DOC)

S1 Table Details of the genetic risk scores for each trait, as previously published. *Previously published beta values obtained from Tyrell et al., 2016.
(PDF)

S2 Table Individual SNP results from meta-analysis of full dataset (PCGC and CHOP combined). *NA = Not available.
(PDF)

S3 Table Comparison of mothers' versus fathers' genetic risk scores (GRS) for adult conditions for the 90th and 75th percentiles (Full dataset). (PDF)

S4 Table Fisher exact test 2 x 2 tables for full dataset of “high” and “low” genetic risk scores. (PDF)
