# Supplementary material for: The association of elevated maternal genetic risk scores for hypertension, type 2 diabetes and obesity and having a child with a congenital heart defect
Source: PLoS One. 2019 May 29;14(5):e0216477. doi: 10.1371/journal.pone.0216477 (PMC6541344; doi:10.1371/journal.pone.0216477)
Supplement: S1 Table — *Previously published beta values obtained from Tyrell et al., 2016. (PDF) [file pone.0216477.s002.pdf]

S1 Table. Details of the genetic risk scores for each trait, as previously published*

Hypertension, type II diabetes and obesity genetic risk score details			
SNPs	Trait-raising allele	*Betas	P-value
	Hypertension SNPs		
rs17367504	A	0.9031	0.79
rs2932538	G	0.3884	0.63
rs13002573	A	0.4160	0.55
rs13082711	C	0.3151	0.63
rs3774372	C	0.0666	0.10
rs319690	T	0.4230	0.89
rs419076	T	0.4088	0.20
rs1458038	T	0.7057	0.48
rs13139571	C	0.3213	0.01
rs1173771	G	0.5041	0.39
rs11953630	C	0.4119	0.73
rs805303	G	0.3756	0.80
rs17477177	C	0.5520	0.26
rs1813353	T	0.5686	0.93
rs4590817	G	0.6457	0.83
rs932764	G	0.4837	0.92
rs11191548	T	1.0952	0.01
rs2782980	C	0.4060	0.37
rs1801253	C	0.5700	0.01
rs7129220	A	0.6186	0.10
rs381815	T	0.5747	0.34
rs633185	C	0.5647	0.03
rs17249754	G	0.9282	0.68
rs3184504	T	0.5976	0.50
rs10850411	T	0.3541	0.14
rs1378942	C	0.6125	0.76
rs17608766	C	0.5564	0.89
rs12940887	T	0.3622	0.39
rs1327235	G	0.3404	0.96
rs6015450	G	0.8964	0.19

Type II diabetes SNPs			
rs10923931	T	0.077	0.47
rs10203174	C	0.131	0.76
rs243088	T	0.068	0.79
rs7569522	A	0.048	0.17
rs1801282	C	0.122	0.06
rs1496653	A	0.086	0.51
rs12497268	G	0.030	0.12
rs11717195	T	0.104	0.07
rs4402960	T	0.122	0.53
rs17301514	A	0.049	0.30
rs6819243	T	0.068	0.03
rs4458523	G	0.095	0.39
rs459193	G	0.077	0.75
rs6878122	G	0.095	0.63
rs7756992	G	0.157	0.28
rs4299828	A	0.039	0.87
rs17168486	T	0.104	0.48
rs849135	G	0.104	0.97
rs17867832	T	0.086	0.76
rs516946	C	0.086	0.11
rs7845219	T	0.058	0.78
rs3802177	G	0.131	0.89
rs10758593	A	0.058	0.24
rs16927668	T	0.039	0.11
rs17791513	A	0.113	0.65
rs2796441	G	0.068	0.2
rs11257655	T	0.068	0.53
rs12242953	G	0.068	0.17
rs12571751	A	0.077	0.52
rs1111875	C	0.104	0.44
rs7903146	T	0.329	0.69
rs2334499	T	0.039	0.07
rs163184	G	0.086	0.44
rs5215	C	0.068	0.91
rs1552224	A	0.104	0.16
rs2261181	T	0.122	0.63
rs7955901	C	0.068	0.81
rs12427353	G	0.077	0.75
rs7177055	A	0.077	0.60
rs12899811	G	0.077	0.65
rs7202877	T	0.113	0.73
rs2447090	A	0.039	0.31

rs8182584	T	0.039	0.50
rs4812829	A	0.058	0.03
	Obesity SNPs		
rs2815752	A	0.13	0.004
rs1514175	A	0.07	0.69
rs1555543	C	0.06	0.82
rs543874	G	0.22	0.49
rs2867125	C	0.31	0.49
rs713586	C	0.14	0.57
rs887912	T	0.10	0.98
rs2890652	C	0.09	0.51
rs13078807	G	0.10	0.24
rs9816226	T	0.14	0.43
rs10938397	G	0.18	0.89
rs2112347	T	0.10	0.60
rs4836133	A	0.07	0.20
rs206936	G	0.06	0.75
rs987237	G	0.13	0.57
rs10968576	G	0.11	0.94
rs4929949	C	0.06	0.96
rs10767664	A	0.19	0.18
rs3817334	T	0.06	0.09
rs7138803	A	0.12	0.53
rs4771122	G	0.09	0.16
rs11847697	T	0.17	0.62
rs10150332	C	0.13	0.09
rs2241423	G	0.13	0.61
rs12444979	C	0.17	0.87
rs1558902	A	0.39	0.98
rs571312	A	0.23	0.53
rs29941	G	0.06	0.04
rs2287019	C	0.15	0.75
rs3810291	A	0.09	0.46

*Previously published beta values obtained from Tyrell et al., 2016.
