# Supplementary material for: The association of elevated maternal genetic risk scores for hypertension, type 2 diabetes and obesity and having a child with a congenital heart defect
Source: PLoS One. 2019 May 29;14(5):e0216477. doi: 10.1371/journal.pone.0216477 (PMC6541344; doi:10.1371/journal.pone.0216477)
Supplement: S2 Table — *NA = Not available. (PDF) [file pone.0216477.s003.pdf]

S2 Table. Individual SNP results from meta-analysis of full dataset (PCGC and CHOP combined)

Hypertension, type 2 diabetes and obesity individual SNP results		
Locus	SNP	p-value
Hypertension individual SNP results		
TBX5-TBX3	rs10850411	0.143
CYP17A1-NT5C2	rs11191548	0.014
NPR3-C5orf23	rs1173771	0.394
EBF1	rs11953630	0.725
ZNF652	rs12940887	0.385
FIGN	rs13002573	0.555
SLC4A7	rs13082711	0.631
GUCY1A3-GUCY1B3	rs13139571	0.0127
JAG1	rs1327235	0.958
CYP1A1-ULK3	rs1378942	0.764
FIGN	rs1446468	NA*
FGF5	rs1458038	0.485
ATP2B1	rs17249754	0.680
MTHFR-NPPB	rs17367504	0.794
PIK3CG	rs17477177	0.262
GOSR2	rs17608766	0.889
ADRB1	rs1801253	0.012
CACNB2(3′)	rs1813353	0.931
ADRB1	rs2782980	0.367
MOV10	rs2932538	0.896
SH2B3	rs3184504	0.501
MAP4 (intron)	rs319690	0.886
ULK4	rs3774372	0.096
PLEKHA7	rs381815	0.336
MECOM	rs419076	0.199
C10orf107	rs4590817	0.832
GNAS-EDN3	rs6015450	0.187
FLJ32810-TMEM133	rs633185	0.030
ADM	rs7129220	0.103
BAT2-BAT5	rs805303	0.796
PLCE1	rs932764	0.923
Type II diabetes individual SNP results		
KLHDC5	re10842994	NA
THADA	rs10203174	0.759
GLIS3	rs10758593	0.239
NOTCH2	rs10923931	0.474

HHEX/IDE	rs1111875	0.444
CDC123/CAMK1D	rs11257655	0.531
ADCY5	rs11717195	0.075
VPS26A	rs12242953	0.169
HNF1A (TCF1)	rs12427353	0.747
PSMD6	rs12497268	0.117
ZMIZ1	rs12571751	0.517
PRC1	rs12899811	0.647
UBE2E2	rs1496653	0.513
ARAP1 (CENTD2)	rs1552224	0.155
KCNQ1	rs163184	0.437
PTPRD	rs16927668	0.106
DGKB	rs17168486	0.480
ST64GAL1	rs17301514	0.299
TLE4	rs17791513	0.652
GCC1	rs17867832	0.759
PPARG	rs1801282	0.064
HMGA2	rs2261181	0.632
DUSP8	rs2334499	0.067
BCL11A	rs243088	0.795
SRR	rs2447090	0.313
TLE1	rs2796441	0.204
SLC30A8	rs3802177	0.890
ZFAND3	rs4299828	0.866
IGF2BP2	rs4402960	0.529
HNF1B (TCF2)	rs4430796	NA
WFS1	rs4458523	0.389
ANKRD55	rs459193	0.754
HNF4A	rs4812829	0.031
ANK1	rs516946	0.113
KCNJ11	rs5215	0.907
MAEA	rs6819243	0.033
ZBED3	rs6878122	0.633
HMG20A	rs7177055	0.598
BCAR1	rs7202877	0.731
RBMS1	rs7569522	0.174
CDKAL1	rs7756992	0.287
TP53INP1	rs7845219	0.785
TCF7L2	rs7903146	0.694
TSPAN8/LGR5	rs7955901	0.811
PEPD	rs8182584	0.502
JAZF1	rs849135	0.973

Obesity individual SNP results

NRXN3	rs10150332	0.092
BDNF	rs10767664	0.182
GNPDA2	rs10938397	0.890
LRRN6C	rs10968576	0.943
PRKD1	rs11847697	0.622
GPRC5B	rs12444979	0.868
CADM2	rs13078807	0.240
TNN13K	rs1514175	0.688
PTBP2	rs1555543	0.816
FTO	rs1558902	0.977
NUDT3	rs206936	0.753
FLJ35779	rs2112347	0.601
MAP2k5	rs2241423	0.615
QPCTL	rs2287019	0.750
NEGR1	rs2815752	0.004
TMEM18	rs2867125	0.492
LRP1B	rs2890652	0.515
KCTD15	rs29941	0.043
TMEM160	rs3810291	0.460
MTCH2	rs3817334	0.092
MTIF2	rs4771122	0.156
ZNF608	rs4836133	0.197
RPL27A	rs4929949	0.960
SEC16B	rs543874	0.488
MC4R	rs571312	0.530
RBJ	rs713586	0.573
FAIM2	rs7138803	0.528
FANCL	rs887912	0.983
ETV5	rs9816226	0.433
TFAP2B	rs987237	0.572

*NA = Not available
