# Supplementary material for: The association of elevated maternal genetic risk scores for hypertension, type 2 diabetes and obesity and having a child with a congenital heart defect
Source: PLoS One. 2019 May 29;14(5):e0216477. doi: 10.1371/journal.pone.0216477 (PMC6541344; doi:10.1371/journal.pone.0216477)
Supplement: S3 Table — (PDF) [file pone.0216477.s004.pdf]

S3 Table. Comparison of mothers' versus fathers' genetic risk scores (GRS) for adult conditions for the 90th and 75th percentiles (Full dataset)

GRS Type	90th %ile OR (95% CI)	90th %ile p-value	75th %ile OR (95% CI)	75th %ile p-value
All (107 SNPs)	GRS>28.08<br>1.74 (1.23, 2.5)	0.001	GRS>27.04<br>1.27 (0.98, 1.64)	0.06
Body Mass Index (30 SNPs)	GRS>4.48<br>1.23 (0.84, 1.79)	0.28	GRS>4.16<br>1.11 (0.85, 1.45)	0.47
Blood Pressure (31 SNPs)	GRS>19.84<br>1.49 (1.04, 2.15)	0.02	GRS>18.97<br>1.09 (0.84, 1.42)	0.51
Type II Diabetes (46 SNPs)	GRS>4.89<br>1.44 (1.00, 2.09)	0.05	GRS>4.67<br>1.02 (0.78, 1.33)	0.89
