# Supplementary material for: The association of elevated maternal genetic risk scores for hypertension, type 2 diabetes and obesity and having a child with a congenital heart defect
Source: PLoS One. 2019 May 29;14(5):e0216477. doi: 10.1371/journal.pone.0216477 (PMC6541344; doi:10.1371/journal.pone.0216477)
Supplement: S4 Table — (PDF) [file pone.0216477.s005.pdf]

S4 Table. Fisher exact test 2 x 2 tables for full dataset of "high" and "low" genetic risk scores

GRS 2x2 tables							
Parent	<95th %ile	>95th %ile	<90th %ile	>90th %ile	<75th %ile	>75th %ile	Total
BMI/hypertension/type II diabetes							
Father	570	29	539	60	450	149	599
Mother	586	53	535	104	450	189	639
Total	1156	82	1074	164	900	338	1238
BMI							
Father	569	30	539	60	454	145	599
Mother	591	48	562	77	472	167	639
Total	1160	78	1101	137	926	312	1238
Hypertension							
Father	570	29	539	60	448	151	599
Mother	588	51	548	91	467	172	639
Total	1158	80	1087	151	915	323	1238
Type II Diabetes							
Father	571	28	542	57	448	151	599
Mother	586	53	555	84	475	164	639
Total	1157	81	1097	141	923	315	1238
